# Effect of Nanoencapsulation on the Antimicrobial and Antibiofilm Activities of Algerian *Origanum glandulosum* Desf. against Multidrug-Resistant Clinical Isolates

**DOI:** 10.3390/nano12152630

**Published:** 2022-07-30

**Authors:** Sarah Bouaouina, Abdelhakim Aouf, Abdelaziz Touati, Hatem Ali, Manal Elkhadragy, Hany Yehia, Amr Farouk

**Affiliations:** 1 Laboratory of Applied Microbiology, Faculty of Life Sciences and Nature, University of Ferhat Abbas, Setif 19000, Algeria; sarah.bouaouna@univ-setif.dz (S.B.); a.aouf@univ-setif.dz (A.A.); 2 Laboratoire d’Ecologie Microbienne, Faculté des Sciences de la Nature et de la Vie, Université de Bejaia, Bejaia 06000, Algeria; abdelaziz.touati@univ-bejaia.dz; 3 Food Technology Department, National Research Center, Cairo 12622, Egypt; hatem.owyean1@gmail.com; 4 Department of Biology, College of Science, Princess Nourah bint Abdulrahman University, P.O. Box 84428, Riyadh 11671, Saudi Arabia; mfelkhadragy@pnu.edu.sa; 5 Food Science and Nutrition Department, College of Food and Agriculture Science, King Saud University, P.O. Box 2460, Riyadh 11451, Saudi Arabia; hanyehia@ksu.edu.sa; 6 Food Science and Nutrition Department, Faculty of Home Economics, Helwan University, Helwan P.O. Box 11611, Egypt; 7 Flavour and Aroma Chemistry Department, National Research Centre, Cairo 12622, Egypt

**Keywords:** multidrug resistant bacteria, *Origanum glandulosum* Desf, nanoencapsulation, antibacterial activity, anti-biofilm activity

## Abstract

The emergence of multidrug-resistant (MDR) bacteria is a danger to public health and exposes patients to high risk, increasing morbidity and mortality worldwide. For this purpose, three months of evaluation of MDR’s prevalence and antimicrobial susceptibility patterns in the military regional university hospital of Constantine from different services and samples was carried out. Among a total of 196 isolates, 35.2% were MDR. The use of essential oils such as *Origanum glandulosum* Desf. as an alternative to antibiotics is attractive due to their rich content of bioactive compounds conferring many biological activities. Also, to overcome the drawbacks of using oils as the hydrophobicity and negative interaction with the environmental conditions, in addition to increasing their activity, encapsulation for the oil was performed using high-speed homogenization (HSH) into nanocapsules and high-pressure homogenization (HPH) into nanoemulsion. Nine volatile constituents were determined using gas chromatography-mass spectrometry analysis (GC-MS) in hydrodistilled oil with thymol, carvacrol, p-cymene, and γ-terpinene as dominants. A dramatic decrease in the major volatile components was observed due to the use of HSH and HPH but generated the same oil profile. The mean particle size of the nanoemulsion was 54.24 nm, while that of nanocapsules was 120.60 nm. The antibacterial activity of the oil and its nanoparticles was estimated on MDR isolates using the disk diffusion, aromatogram, and broth microdilution methods. Consistent with the differences in volatile constituents, the oil exhibited a higher antibacterial activity compared to its nanoforms with the diameters of the inhibition zone against *E. coli* (20 mm), *S. aureus* (35 mm), and *A. baumannii* (40 mm). Both formulations have shown relatively significant activity against the biofilm state at sub-inhibitory concentrations, where nanoemulsion was more potent than nanocapsules. The results obtained suggested that nanoformulations of essential oils are strongly recommended for therapeutic application as alternatives to antibiotics.

## 1. Introduction

Antibiotics have been very efficient in treating and preventing infectious diseases caused by pathogenic bacteria since the discovery of the first antibiotic in the last three decades [1]. Pathogenic bacteria have become highly resistant to most antibiotics used in human and animal medicine during the previous three decades, and resistant clones have spread worldwide for many reasons, such as limiting uptake of a drug and biofilm formation [2]. Microorganisms use biofilms to store nutrients and endure harsh environmental conditions, which are critical for their existence. Biofilm development on human mucosa is responsible for about 60% of human illnesses [3]. Multidrug-resistant (MDR) organisms are microorganisms that have developed resistance to particular antibiotics and can no longer be controlled or killed by these drugs. Hospitals, nursing homes, and long-term care institutions are the most common places to find multidrug-resistant microbes. They commonly affect children, the elderly, or the terminally ill and can result in serious infections. MDR bacteria’s proliferation into the community is a critical issue, as it is linked to higher morbidity, death, healthcare expenses, and antibiotic use. Antibiotic, antibacterial, and antimicrobial resistance are used to describe this [4].

An attractive alternative for synthetic antibiotics is the naturally occurring plant-derived extracts and essential oils that can prevent biofilm development. Essential oils are hydrophobic, extracted from aerial parts of aromatic plants, and are promising natural ingredients due to their preservative and antimicrobial effects. Szczepanski and Lipski [5] analyzed the inhibiting effects of thyme, oregano, and cinnamon essential oil at sublethal concentrations on biofilm formation of three biofilm-forming bacterial strains. These strains of the genera *Acinetobacter*, *Sphingomonas,* and *Stenotrophomonas* were isolated from authentic biofilms in the food industry. It was reported by Kim and collaborators that biofilm formation by *E. coli* 0157: H7 is inhibited by essential oils and eugenols at sub-inhibitory concentrations without affecting planktonic cell growth [6]. *Origanum glandulosum* (Desf.) is an endemic species in north Algeria. It was reported that its essential oil has potent antibacterial activity against MDR *E. coli* isolates [7].

However, due to their poor dispersibility in hydrophilic media and susceptibility to environmental conditions, direct applications of essential oils in foods or pharmaceuticals may be limited. Essential oils can be encapsulated in delivery systems to optimize their solubility, stability, and controllable release, extending their potency over time [8]. The formulation of nanoemulsions boosted the surface-to-volume ratio of these nano-sized delivery systems, resulting in improved reactivity and effective absorption through cells and regulated release and targeting of bioactive substances at the site of action [9].

The present study focuses mainly on assessing the incidence of MDR in hospital settings, evaluating the antibacterial activity of essential oil extracted from *Origanum glandulosum* Desf. growing wild in Bejaia, the north of Algeria, investigating the chemical composition and then estimating the influence of nanoencapsulation and nanoemulsion using high-speed homogenization (HSH) and high-pressure homogenization (HPH) on antibacterial and antibiofilm activities. In this study, the investigation of safe materials of botanical origin represented a flourishing opportunity for applying nanoparticles of essential oils in some clinical situations to treat or prevent infections with MDR strains.

## 2. Materials and Methods

### 2.1. Study Design

A three-month prospective study was conducted at the central microbiology laboratory in the regional military university hospital of Constantine. A total of 323 specimens were analyzed during this period; all hospitalized patients were involved except those of pediatric service. These specimens were collected from different sources (urines, pus, cerebrospinal liquid (CSL), and hemocultures).

### 2.2. Identification of Clinical Isolate

Collected pure isolates from different specimens were identified according to standard culture techniques based on morphological and biochemical characterization. Gram-positive cocci occurring in clusters were subjected to growth on mannitol salt agar, catalase [10], and coagulase tests [11]. Gram-negative bacilli isolates were purified on selective media and identified to the species using a qualitative micro method employing conventional and chromogenic substrates to identify clinically important selected oxidase-negative. Identification kit RapID™ ONE system tests and Remel™ RapID™ ERIC™ Software (Thermo Scientific, Waltham, MA USA) were used to identify isolates according to the recorded database.

### 2.3. Antibiotic Susceptibility Test

The study of the susceptibility of bacteria to antibiotics was carried out using the Kirby-Bauer test. The quality control of purchased antibiotic disks (Oxoid, Basingstoke, Hampshire, UK) was performed using reference strains (*Staphylococcus aureus* ATCC 25923, *Escherichia coli* ATCC 25922, and *Pseudomonas aeruginosa* ATCC 27853) on Muller Hinton Agar at 37 °C for 18 h. Zone diameters were interpreted according to CLSI breakpoint criteria (2014). Eighteen different antibiotic disks (Penicillin G (P10), Oxacillin (OX5), Cefoxitin (FOX30). Spiramycin (SP100), Clindamycin (DA2), Pristinamycin (PT15), Lincomycin (L15), Erythromycin (E15), Rifampicin (RD5), Vancomycin (VA30), Teicoplanin (TEC30), tetracycline (TE30), Doxycycline (DO30), Amikacin (AK30), Ofloxacin (OFX5), Fusidicacid (FD10), Chloramphenicol (C30) Gentamicin (GN10) were used to evaluate the susceptibility of *S. aureus* to antibiotics. Sixteen different antibiotics were applied for *Enterobacteriaceae* members; Ampicillin (AMP10), Amoxicillin (AMX25), Amoxicillin/clavulanate (AMC10), Ticarcillin (TIC75), Piperacillin (PRL100), Cefazolin (CF30), Cefotaxime (CTX30), Ceftriaxone (CRO 30), Imipenem (IMP10), Gentamicin (GN10), Ofloxacin (OFX5), Ciprofloxacin (CIP5), Colistin (CT10) Nalidixic acid (NA30). Trimethoprim/Sulfamethoxazole (SXT1.25/23.75), and Amikacin (AK10). While fifteen antibiotics were tested for *P. aeruginosa and A. baumannii*; Ticarcillin (TIC75), Ticarcillin-clavulanate (TCC75), Piperacillin (PIP100), Piperacillin-clavulanate (TZP110), Ceftazidime (CAZ30), Aztreonam (ATM30), Gentamicin (GN10), Netilmicin (NET30), Ciprofloxacin (CIP5), Levofloxacin (LEV5), Colistin (CT10), Trimethoprim/Sulfamethoxazole (SXT1.25/23.75), Amikacin (AK30), and Rifampicin (RD5).

### 2.4. Phenotypic Detection of MDR Strains

Isolates were selected based on resistance against third-generation cephalosporins (CTX and CAZ) for screening extended-spectrum beta-lactamases (ESBLs) and cephalosporinases production. ESBLs production was detected using a double-disk synergy test (DDST), enhancing the inhibition zone (synergy), indicating a positive test. Positive ESBLs were confirmed by a double disk diffusion test (DDDT) [12]. Phenotypic detection of AmpC cephalosporinases was performed using cloxacillin as an inhibitor (200 μg/mL), as described by Tan et al. [13]. Screening for Methicillin-resistant *S. aureus* (MRSA) was performed using the following antibiotic disks Ox (5µg) and Fox (30 µg) [14].

### 2.5. Screening of Biofilm Production

Detection of biofilm production by selecting five clinical isolates in vitro can be monitored as described by Saxana et al. [15] using a standard crystal violet test. Isolates were grown in brain heart infusion (BHI) Broth at 37 °C for 24 h. The 96 well polystyrene microplate was filled with 100 µL of sterile BHI Broth and 100 µL of the bacterial inoculums adjusted to 0.5 McFarland; the sterile BHI Broth was used as a negative control, and inoculated microplates were then incubated for 48 h at 37 °C. After incubation, the well content was removed and rinsed three times with sterile physiological water, then dried for 15 min at room temperature. The microplates were stained with 200 µL of crystal violet at 0.2% for 15 min, followed by washing with sterile saline water three times and drying for one hour. Retained stain in adherent cells with their formed biofilm was resolubilized by adding 150 µL of 30% acetic acid, and the optical density (OD) was measured at 630 nm using a microplate reader. Results obtained were used to classify tested isolates in comparison with control into weak biofilm producers (OD  ≤  2*ODc), moderate biofilm producers (2*ODc  ≤  OD  ≤  4*ODc), and strong biofilm producers (4*ODc  ≤  OD) [15].

### 2.6. Plant Material and Extraction

*O. glandulosum* growing wild was collected in flowering times (June 2019) from the region of Bejaia. Identification was performed by an ecologist at the biology laboratories at the Faculty of Sciences of Bejaia University. The plant’s aerial part (leaves, flowers, and stems) was dried in the dark at room temperature (25–26 °C); essential oil was obtained by steam distillation for 3 h and stored in a brown glass bottle at 4 °C until their use.

### 2.7. Nanoencapsulation and Nanoemulsion Formulation

Nanoencapsulation of *O. glandulosum* was obtained by high-speed homogenization (HSH) using sodium alginate, tween 20, and distilled water. Nanoemulsions were prepared using a High-Pressure Homogenization (HPH) technique; emulsions were obtained by mixing a solution consisting of 1 g oil and 1% of Tween 20 in 100 mL of deionized water using an Ultra Turrax T25 (IKA Labortechnik, Staufen, Germany) at 24,000 rpm for 10 min. The emulsion was created by mixing the solution in a high-speed homogenizer at 18,000 rpm for 20 min. The temperature was maintained at 25 °C [16].

### 2.8. Bacterial Strains

The effectiveness of the essential oils was evaluated against nine bacterial species involved in food poisoning and infectious diseases. *Pseudomonas aeruginosa* ATCC2525, *Staphylococcus aureus* ATCC6538P, *Klebsiella pneumoniae* ATCC700603, *E. coli* ATCC8739, and five multiresistant clinical isolates (*Escherichia coli* ESBL, *Klebsiella pneumoniae* ESBL, *Acinetobacter baumannii* MDR, *Serratia marcescens* ESBL and MRSA), cell cultures maintenance of all of the strains was carried out in BHI broth and incubated at 37 °C for 24 h to obtain fresh cultures.

### 2.9. Antimicrobial Activity

#### 2.9.1. Agar Diffusion Method

The antimicrobial activity was determined by the method described previously [17]. Briefly, the MH agar plate was inoculated with adjusted bacterial suspension (10^6^ CFU/mL), then diluted essential oils in (two-fold serial dilution) dimethyl sulfoxide (DMSO) (20 μL) were poured in wells of 6 mm diameter, and DMSO was used as a negative control. After that, plates were refrigerated at 4 °C for two hours to allow the essential oils to diffuse into agar, followed by incubation at 37 °C for 24 h; the antibacterial activity of each sample was evaluated by measuring the zone of inhibition diameter expressed in millimeters (mm). The assays were performed in triplicate [18].

#### 2.9.2. Broth Microdilution Assay

The analysis of minimum inhibitory concentration (MIC) was carried out by the broth microdilution method using 96 well microplates. Essential oils were dissolved in DMSO then two-fold serial dilution using BHI broth was made to form (5–0.04%) v/v, each well containing 50 µL of the essential oil and 50 µL of a bacterial suspension at a final concentration of 10^6^ CFU/mL. Bacterial suspensions were prepared from a 24 h culture in BHI broth. The microtiter plates were sealed with parafilm tape and incubated at 37 °C for 24 h. The negative control well consisted of 100 μL of BHI Broth; the positive well consisted of 100 μL of BHI broth with a bacterial suspension without essential oil. MICs were estimated using Tetrazolium chloride (2,3,5-triphenyl-2H-tetrazolium chloride TTC) as an indicator of viability. The minimum bactericidal concentration (MBC) is determined by subculturing 10 μL of broths used for MICs measurement onto fresh agar plates, then incubating at 37 °C for 24 h. The MBC was identified as the concentration on which no colony growth was observed [19].

### 2.10. Chemical Composition of HD Oil and Its Nanoparticles

Gas chromatography-mass spectrometry (CG-MS) analyzed hydrodistilled oil (HD) and its nanoparticles; separation was realized on a Trace GC Ultra Chromatography system (Thermo Scientific, Waltham, MA, USA) equipped with an ISQ-mass spectrometer (Thermo Scientific, Waltham, MA, USA) with a 60 m × 0.25 mm × 0.25 μm-thick TG-5MS capillary column (Thermo Scientific, Waltham, MA, USA). The column separation was programmed at 50 °C with a holding time of 3 min, and then the temperature was increased at a rate of 4 °C per min to 140 °C with a holding time of 5 min. After that, the temperature increased from 6 °C per minute to 260 °C for a 5-min isothermal holding time. The injector temperature was 180 °C, the ion source temperature was 200 °C, and the transition line temperature was 250 °C. The carrier gas was helium with a constant flow rate of 1.0 mL/min. The mass spectrometer had a scan range from 40–450 *m/z*, and the ionization energy was set at 70 Ev. The identification of compounds was based on matching profiles with the MS computer library (NIST library, 2005 version) and comparison with raw compounds and published data 28. The percentage of the identified constituents was calculated from the GC peak areas. Kovat’s index was calculated for each compound using the retention times of a homologous series of C_6_–C_26_ n-alkanes and by matching with the values reported in the literature [20,21].

### 2.11. Particle Size and z-Potential

The prepared particles were analyzed for their particle size and size distribution in terms of the average volume diameters and polydispersity index by photon correlation spectroscopy using the particle size analyzer Dynamic Light Scattering (DLS) (Zetasizer Nano ZN, Malvern Panalytical Ltd., Grovewood Road, Malvern, UK) at a fixed angle of 173 °C at 25 °C. Samples were analyzed in triplicate. DLS measures the Brownian motion of nano-sized droplets and relates this movement to an equivalent hydrodynamic diameter (nm). Average droplet size, size distribution curves, and polydispersity index were used to characterize oil droplet dispersion in nanoemulsions. The electrophoretic mobility of oil droplets also reported as z-potential, was measured by the same equipment mentioned above. It determines the surface electrical charge of the droplets dispersed in the continuous phase [22].

### 2.12. Antimicrobial Assay

#### 2.12.1. Agar Diffusion Assay

The nanoemulsion and nanoencapsulation antibacterial activity were evaluated against the previously mentioned pathogenic bacteria using the agar diffusion method as described before.

#### 2.12.2. Broth Microdillution Assay

The evaluation of MICs of the nanoemulsion and nanoencapsulation was carried out on 96 well microplates, and the formulation was diluted in BHI to obtain a concentration range from (500 µL/mL to 3.9 µL/mL) the bacterial inoculum was prepared in BHI and adjust to a concentration of 10^6^ UFC/mL the microplate is incubated at 37° for 24 h. The minimum bactericidal concentration (CMB) was obtained by transferring the contents of the well to a solid medium

### 2.13. Effect of Nanoformulations and Essential Oil of O. glandulosum on Biofilm Formation

The biofilm inhibition assay investigated the possibility of *O. glandulosum* essential oil nanoformulations to prevent initial cell attachment. The antibiofilm activity was performed using a sterile 96-well polystyrene microplate, each well was filled with 100 µL of essential oil at the sub-inhibitory concentration (to be added in experimentation) and 100 µL of the bacterial inoculum adjusted to 0.5 Mc Farland (corresponding to 10^5^ CFU/mL) the plate was incubated at 37 °C for 48 h. The bacterial culture without nanoformulations was considered as a positive control, while the broth was negative. After incubation the contents of each well was removed, and the microplate was rinsed three times with sterile physiological saline 0.9% and dried at 60 °C for 45 min then wells were stained with 200 µL of crystal violet 0.4% and incubated at room temperature for 15 min; the microplate was rinsed three times with sterile physiological saline 0.9%. Then 200 µL of acetic acid at 30% prepared with ultra-pure water was added to each well, the absorbance measurement performed at 595 nm and the inhibition percentage were calculated by the following formula [23]:

% Inhibition Percentage = ((OD negative control-OD test)/(OD negative control)) ∗ 100(1)

## 3. Results and Discussion

### 3.1. Volatile Constituents of O. glandulosum Desf. Oil and Its Nanoparticles

The GC-MS analysis identified nine compounds representing 99.99% of the total oil content (Table 1). Thymol (48.52%), carvacrol (16.13%), p-cymene (27.56%), and γ-terpinene (5.59%) were the predominates in the HD oil. Consequently, the presented oil belongs to the thymol-carvacrol chemotype. Previous studies on essential oil extracted from the same species, collected from Setif and Bejaia, were in line with the present study’s results and the same aroma profile [20,24]. However, some quantitative differences could be observed in both p-cymene and γ-terpinene, which may be attributed to the differences in environmental conditions or agronomical activities which affected the oil composition [25].

A dramatic change could be observed in the composition of the oil nanocapsules compared with HD oil, where 22 compounds were identified, accounting for 96.04% of the total volatiles (Table 1). Compared to HD oil, the same aroma trend could be observed but with remarkable quantitative differences in the predominates. A large decrease was observed in thymol (39.47%), carvacrol (7.07%), p-cymene (18.58%), and γ-terpinene (4.52%). On the other hand, new non-oxygenated isomers such as α-terpinene (2.39%) and many oxygenated terpenes, especially alcohol such as linalool (2.97%), borneol (2.43), and terpinen-4-ol (2.95%) were formed as a consequence of the nanoencapsulation by HSH. In the same context, thymol (51.42%), carvacrol (9.96%), and p-cymene (19.78%) were the major volatile components identified in the nanoemulsion of the HD oil, while γ-terpinene was determined in a low concentration (0.77%) (Table 1). Notably, the concentration of non-oxygenated volatile compounds in the nanoemulsion is lower than in nanocapsules. In contrast, oxygenated terpenes showed an increase in the nanoemulsion compared to nanocapsules (Table 1). The previous findings seem to be related to the difference in homogenization conditions and the amount of energy applied.

Most studies deal with the encapsulation of oils or flavors, focusing on the physical stability and biological activity of microparticles or nanoparticles but not on the changes that may occur in the volatile contents of the encapsulated oils. According to Donsì et al. [26] and Chang et al. [27], the formulation based on energy-intensive techniques such as HSH and HPH may lead to Ostwald ripening, flocculation, or coalescence of the emulsion with changes in its physical stability and biological activity. For example, Weiss et al. [28] and Aouf et al. [29] reported a reduction in nanoemulsions’ antimicrobial and antioxidant activities compared to raw active ingredients. Consistent with our findings, HPH and HSH techniques decompose active constituents of essential oils, particularly p-cymene, terpinenes, carveol, carvacrol, and others [26]. In addition, thymol and carvacrol concentrations were increased in the nanoemulsion of Algerian *Saccocalyx satureioides* Coss. et Durieu [29] on the expense of borneol and α-terpineol and vice versa [20].

### 3.2. Particle Size and z-Potential of Nanoparticles

**Figure 1:**
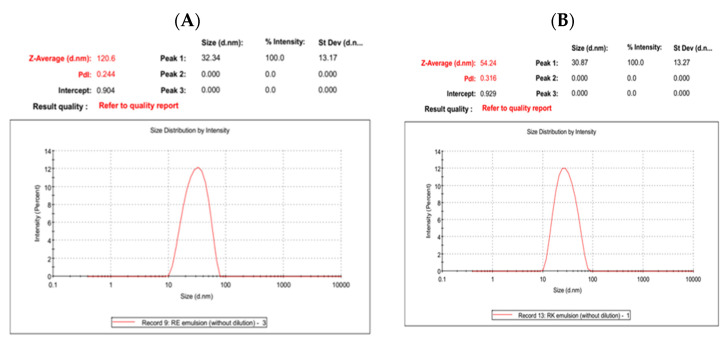
Particle size distribution of (**A**) *O. glandulosum* Desf. oil nanocapsules and (**B**) nanoemulsion.

The mean particle size of the *O. glandulosum* Desf. oil nanoemulsion was recorded at 54.24 ± 1.29 nm, confirming the ultra-fine size (<100 nm). In the same context, the zeta potential mean was −26.2 ± 2.6 mV. Howevevr, the mean of poly dispersibility index (PDI) was equal to 0.296 ± 0.09 (Table 2). These values reflected the better stability of the formed emulsion. In the same context, *O. glandulosum* Desf. oil nanocapsules showed a larger particle size (120.60 ± 18.37 nm) with a lower z-potential (−15.5 ± 0.32 mV) and PDI value (0.244 ± 0.05) compared to the nanoemulsion (Table 2). The above differences are related to the processing conditions, where more intensive energy is applied to prepare nanoemulsion. The results in Figure 1 indicated that both types of nanoemulsion particle size were a monomodal size distribution pattern.

Danaei et al. [30] showed the approximate particle size range between 10 nm–20 µm for drug deposition in various body organs based on different dosage forms and routes of administration. A PDI of 0.3 and less is acceptable in drug delivery applications using lipid-based carriers such as liposome and nanoliposome formulations and implies a homogeneous population of phospholipid vesicles [30]. The zeta potential plays a vital role in the physical stability of emulsions, with a higher zeta potential (positive or negative) indicating more stable emulsions [31]. Therefore, the z-potential value of the oil nanoemulsion suggests more stability than the microcapsules (Table 2).

### 3.3. Clinical Bacterial Isolates According to Specimens

**Figure 2:**
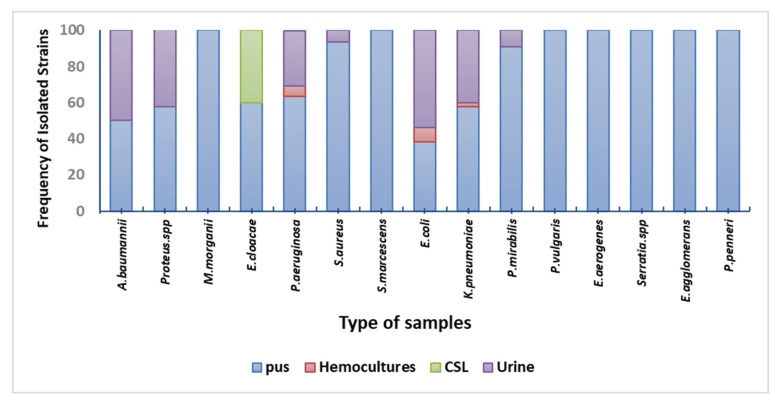
Distribution of clinical bacterial isolates according to specimens.

Patients from whom specimens were obtained were hospitalized at the military regional university hospital of Constantine. A total of 196 bacterial isolates were recovered from 323 different specimens. Analysis of individual positive cases by species using phenotypic methods showed the predominance of highly virulent pathogens, with a high frequency of isolation of *Enterobacteriaceae* (64.79%) followed by non-fermentative gram-negative *bacilli* (19.38%) and *Staphylococcus aureus* (15.81%). Most samples were collected from pus (57.65%) and urine (29.59%) (Figure 2). The above results agree with Kibret and Abera [32], who reported that *Enterobacteriaceae* were predominant at Bahir Dar Regional Health Research Laboratory, Ethiopia, followed by *Pseudomonas spp.* and *S. aureus*. Also, Hawser and collaborators [33] reported the predominance of *Enterobacteriaceae* members (*E. coli, K. pneumoniae*) and *P. aeruginosa* from intra-abdominal infections in 19 hospitals within the United States. A similar study in Malawi hospital reported that pus was the main source of microorganisms (69.3%), and adults accounted for most isolates (88.3%). *S. aureus* (34.7%), *Klebsiella* species (17.4%), and *Proteus* species (11.4%) were the most common isolates. Most *S. pneumoniae* isolates (60.3%) came from the cerebral fluid primarily obtained from kids (88.2%). Except for ciprofloxacin, most bacteria resisted all commonly used antimicrobials [34].

Antimicrobial susceptibility patterns often differ between geographical regions, populations, hospital types, and units [35,36]. The results of the present study are particularly alarming. It should emphasize the need for the implementation of a surveillance program nationwide. In addition, specific adjustment of systemic antibiotic therapy is required because the emergence of pathogenic bacteria poses a serious threat to public health.

### 3.4. Analysis of Clinical Bacterial Isolates According to the Department

**Figure 3:**
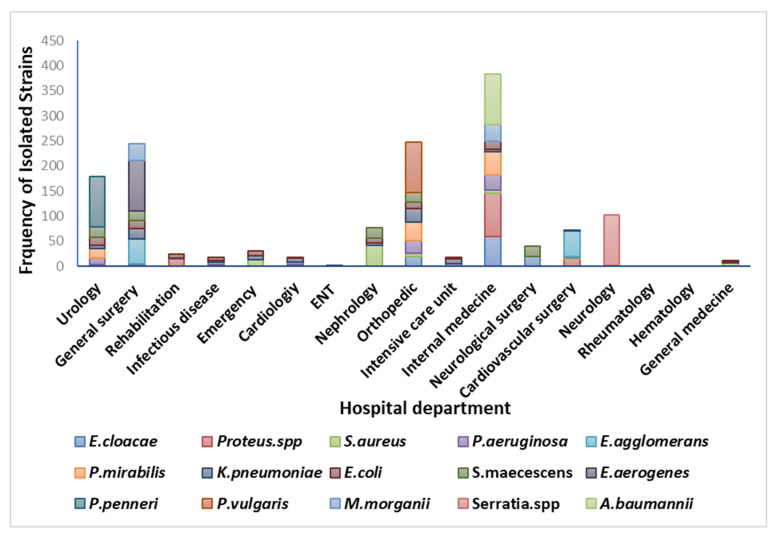
Distribution of isolates according to hospital departments.

Analysis of the prevalence of bacterial isolates according to hospital departments showed that most pathogens were isolated from specimens issued from the department of internal medicine (39.89%) and the department of orthopedics (31.33%) (Figure 3). This is mainly due to admissions of patients with a wide variety of infections at internal medicine unit and nature of army practices for the department of orthopedics. Thus, effective strategies in these areas are strongly recommended. However, most studies report the general surgery and intensive care unit as the primary high-risk department since the utilization of invasive procedures, including catheterization, central lines, and mechanical ventilation, are a few predisposing variables linked to the higher infection rates among hospitalized patients [37].

### 3.5. Antibiotic Resistance Frequency of MDR

Susceptibility to a range of commonly used antibiotics for clinical isolates showed that all isolates were resistant to most tested antibiotics, except for imipenem (IMP) and amikacin (AK). Gentamicin (GN), ofloxacin (OFX), ciprofloxacin (CIP), and colistin (CT) also showed potential activity against *E. cloacae* and *E. aerogene*. At the same time, OFX, CT, and Levofloxacin (LEV) are effective against *A. baumannii*, *K. pneumoniae*, and *M. morganii,* respectively. Some antibiotics may have an intermediate inhibition effect on some MDR isolates such as CIP, while others such as CT have a powerful impact on MDR *E. coli* (Table 3).

In general, many authors have reported an increase in the prevalence of MDR pathogens in recent years. According to Vasireddy et al. [38], 51 multidrug-resistant clinical isolates of *Burkholderia cepacia* complex were resistant to tobramycin and colistin, while a majority were resistant to all other tested antibiotics except for meropenem (only 22% were resistant). In the same context, Ansari et al. [39] reported that all examined *E. coli* isolates were resistant to amoxicillin-clavulanic acid, and 77% were resistant to ciprofloxacin, whereas a few isolates (7%) were resistant to imipenem, and all isolates were sensitive to colistin.

*Staphylococcus aureus* one of the most commonly identified pathogens in hospitals and communities, has proven to be particularly adept at generating antibiotic resistance, limiting therapeutic choices to treat infections caused by this bacterium. (MRSA) and *S. aureus* with lower susceptibility to vancomycin (VA) have gained popularity [40]. In the present study, VA showed superior in vitro activity compared with the other antibiotics against the MRSA isolates (Table 4). Meanwhile, Pristinamycin (PT), Spriramycin (SP), and Clindamycin (DA) have retained strong effectiveness with a resistance percentage of 7 to 14% against MRSA (Table 4). For two decades, *S. aureus* resistant to methicillin and vancomycin has become one of the leading causes of nosocomial infections in hospitals worldwide. The easy spread of MDR *S. aureus* (MRSA, resistant to VA) via close contact with infected people, contaminated objects, and human carrier require an urgent strategy to reduce the transmission of this potential pathogen in care settings [41].

### 3.6. Phenotypic Detection of MDR Isolates

Analysis of susceptibility results showed that (35.20)% were MDR. The phenotypic study of MDR showed that the predominance was for *Enterobacteriaceae* producing ESBL (27.47%), this value agrees with that obtained in Tunisia (30.8%) and Egypt (38.5%) [42], followed by MRSA (7.69%) and Gram-negative non-fermenting bacilli (*P. aeruginosa and A. baumannii*) resistant to third-generation cephalosporins (2.74%), and the lowest phenotype frequency was for *Enterobacteriaceae* producing cephalosporinases (2.04%). ESBLs are generating *Enterobacteriaceae* isolates, mainly *E. coli* and *Klebsiella pneumoniae*, which have spread quickly in most countries. When found, ESBLs are clinically significant and show that the use of the proper antibacterial drugs is required. Multi-resistance, however, frequently makes choosing an antibiotic difficult [43].

### 3.7. Antibacterial Activity

The antibacterial activity of *O. glandulosum* HD oil and its nanoparticles was estimated on multidrug resistant isolates using two methods: disk diffusion method, (aromatogram) and broth microdillution method (MIC, and MBC), as shown in Table 5. *O. glandulosum* essential oil showed significant activity against all Gram-positive and negative isolates except for *Pseudomonas aeruginosa*. In an aromatogram, diameters of inhibition zone for *E. coli* was 20 mm), *S. aureus* 35 mm, and *Acinetobacter baumannii* 40 mm. The results obtained by MIC and MBC were similar to that obtained by aromatogram (Table 5).

Previous findings agreed with Nabti et al. [7], who studied the antibacterial activity of *O. glandulosum* oils obtained from different Algerian locations. The antibacterial activity of the EOs against six uropathogenic MDR and two standard *E. coli* strains revealed a potential activity against all tested strains, showing similar inhibition zone diameters as well as MIC and MBC values. At a dilution of 1/10, the most powerful EO had the highest percentage of carvacrol (59.6%), with diameters ranging from 12 to 24.5 mm. Similarly, Béjaoui et al. [44] showed a potential activity for Tunisian *O. glandulosum* EOs against a reference *E. coli* ATCC 8739 strain at various phenological stages. When several Algerian *O. glandulosum* EOs were tested against *E. coli* ATCC 25922 and three other clinical strains (E1, E2, and E3), similar findings (excellent growth inhibition) were observed [45,46]. Thymol and carvacrol are phenolic terpenes characterized by their antibacterial activities by damaging the structure and function of the cytoplasmic membrane of the MDR bacteria [47]. Other major components of *O. glandulosum* HD oil, such as *p*-cymene, may synergize the phenolic monoterpenes effect due to its highly hydrophobic properties, which causes swelling of the cytoplasmic membrane, making the transport of effective active compounds across the lipid bilayer more accessible [48].

The antibacterial activity of the *O. glandulosum* oil nanoemulsion and the nanocapsules was evaluated using the agar well diffusion method (Table 5). The inhibition zones ranged between 11 and 20 mm against tested reference strains (*Staphylococcus aureus* ATCC6538P, *E. coli* ATCC25953, K. *pneumoniae* ATCC700603) and clinical *A. baumannii* isolate. Susceptibility of MDR isolates to nanoformulations showed that nanocapsules were more efficient than nanoemulsion. The latter was not active on *E. coli* ESBL, *K. pneumoniae* ESBL, *S. marcescens* ESBL, and *P. aeruginosa* ATCC27853. At the same time, nanocapsules were not active only on *K. pneumoniae* ESBL and *P. aeruginosa* ATCC27853. Microdilution and diffusion methods evaluated MIC and MBC for nanoformulation preparations for all susceptible strains. MIC values ranged from 31.25 µL/mL to 62.5 µL/mL for nanoemulsion and 31.25 µL/mL for nanocapsules. MBCs ranged from 31.5 to 250 µL/mL for both formulations. To the best of our knowledge, the present study described for the first time the effect of nanoencapsulation on the antibacterial activity of the *O. glandulosum* oil.

The nanoencapsulation using intensive-energy techniques is responsible for the changes in aroma profiles discussed above and consequently for the antibacterial activities. The higher concentrations of thymol, carvacrol, and p-cymene are responsible for HD oil’s potential activity compared to nanoparticles. On the other hand, nanocapsules that showed higher non-oxygenated monoterpenes compared to nanoemulsions exhibited a higher antibacterial activity. Increasing the concentration of non-oxygenated terpenes against oxygenated ones may be responsible for better bioactivity. For example, *Satureja thymbra* Growing Wild in Libya showed excellent antimicrobial activity, where γ-terpinene (39.23%), thymol (25.16%), p-cymene (7.17%), and carvacrol (4.18%) constitute the predominates of the HD oil [49]. γ-Terpinene represents one of the major nanocapsules components and is found as a nanoemulsion minor. The antimicrobial activity of essential oils may be altered by synergistic and antagonistic effects between some components. Generally, nanoencapsulation may negatively affect the volatile and active constituents of EOs due to using intensive-energy techniques, consequently reducing the biological activity of the nanoparticles compared to HD oil [20].

### 3.8. Antibiofilm Activity

**Figure 4:**
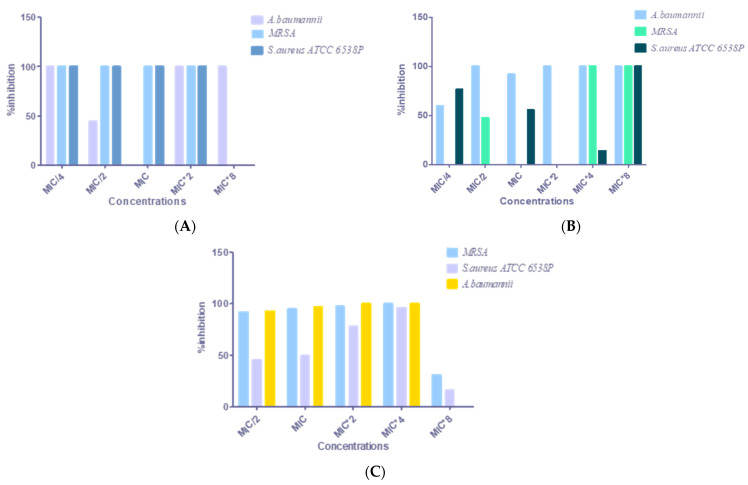
The inhibitory effect of *O. glandulosum* essential oil and their nanoformulations on biofilm formation by pathogenic bacteria: (**A**) effect of nanoemulsion, (**B**) effect of nanocapsules, and (**C**) effect of HD oil.

Chronic and recurrent microbial infections in the human body are frequently due to bacterial biofilm (approximately 80%). Microbial cells within biofilms resist significantly antibiotics, 10 to 1000 times more than the planktonic cells, making their eradication difficult. Recently, a great deal of effort has been directed toward the problem of fighting against biofilm formation by pathogenic bacteria [50]. In this study, the detection of biofilm production ability by selected isolates according to MDR criteria classifies producers into two groups (moderate and strong). *A. baumannii, E. coli*, and *K. pneumoniae* were highly biofilm producers. However, MRSA and *S. aureus* ATCC*6538P* was moderately biofilm producer. Results obtained on the effect of different concentrations of the oil on pathogenic strains (Figure 4) showed that concentrations MIC*2 and MIC*4 corresponded respectively to 0.31% and 0.62% for *S. aureus* ATCC6538P and MRSA, and 0.15% and 0.31% for *A. baumannii* showed better antibiofilm activity against the three strains tested, whereas the MIC*8 shows a low antibiofilm activity. Nostro et al. [51] demonstrated that treating *S. aureus* strains with high concentrations of carvacrol (above the MICs) led to a low mutation frequency. In the same context, Rodrigues et al. [52] showed that exposure to sub-MICs of essential oregano oil induces biofilm formation in *S. aureus.*

On the other hand, the impact of nanoemulsion was higher than that of nanocapsules, where the inhibition percentage was 100% for all of the strains except for *A. baumannii* (55%). In contrast, the best effect on using nanocapsules as antibiofilm was at the concentration MIC*8, whereas a low concentration stimulated biofilm formation with 0% inhibition for *S. aureus* ATCC 6538 P and MRSA. These findings are in agreement with those obtained by Nostro et al. [51], who mentioned that contact of *S.aureus* strain with low concentrations of carvacrol (sub-MICs) resulted in an adaptative response, and was associated with a decrease in susceptibility to carvacrol and an increase in biofilm formation, sub-MICs of phenolic compounds (e.g., citronellol, vanillin, 4-hydroxybenzoic) found in EOs can exert biofilm enhancing effects on some bacterial taxa [53,54].

In line with the above findings, Sousa et al. [55] showed the synergistic antimicrobial effects of carvacrol, α-terpinene, γ-terpinene, ρ-cymene, and linalool against *Gardnerella* species biofilms. While α- and γ-terpinene had no impact, ρ-cymene was able to reduce 30% of the overall biomass, but its impact on bacterial culturability was negligible. Furthermore, although carvacrol + ρ-cymene had a greater synergistic effect against planktonic cultures, carvacrol + linalool was the most active combination against biofilms. This last combination was more efficient (50%) than the entire EO (30%) in lowering the total biomass of the biofilm and also shows that antimicrobial sensitivity in biofilm cultures cannot be predicted using standardized MIC measurements, consistent with our findings [56,57]. In addition, the nanoemulsion of *Thymus daenensis* oil, which have a similar aroma profile to *O. glandulosum* oil, showed a considerable anti-biofilm activity at a sub-lethal dose (56.43% inhibition in 1/2 MIC concentration) after 24 h of incubation compared to 20.65% for the pure oil [58].

Average size and z-potential have been reported to potentially impact the antimicrobial activity, which explains the higher activity of nanoemulsion as antibiofilm compared to nanocapsules. For example, Silva et al. [59] showed an increase in the antimicrobial activity of eugenol, thymol, geraniol, and menthone as nanoemulsions upon homogenization from 7000 rpm to 12,000 rpm. This occurrence is in line with what has been documented in the literature. When Mutlu-Ingok et al. [60] tested antimicrobial activity with nanoemulsions of essential oils with various z-average values, they discovered an inverse association between suspended particle average size and antibacterial action. The lower the z-average value, the higher the nanoemulsion’s activity. In studies utilizing cinnamaldehyde nanoemulsions against *E. coli*, *S. aureus*, *L. monocytogenes*, and *S. enterica*, Otani et al. [61] found similar results. Treatments with smaller particle sizes produced the most inhibitory effect because nanoparticles increase the sample’s contact surface with the microbe, making bioactive substances more accessible and allowing them to pass through cell membranes [62]. The application of nanotechnology provides a plethora of promising and forward-looking strategies for boosting conventional antibiotic therapies and countering the onset of the enormous burden of multidrug resistance. The significant effect of *O. glandulosum* essential oil nanoformulations on biofilm formation at low concentrations (sub-MICs) gives rise to an attractive alternative to reduce the impact of biofilm formation on human health significantly.

## 4. Conclusions

The use of intensive-energy encapsulation techniques has affected the volatile composition of the oil and its nanoparticles, leading to a difference in both antibacterial and antibiofilm activities tested. In line with the higher concentrations of carvacrol and p-cymene, the *O. glandulosum* oil exhibited a higher antibacterial activity than its nanoforms. Both formulations have shown relatively significant activity against biofilm state at sub-inhibitory concentrations, where nanoemulsion was more potent than nanocapsules due to the higher thymol concentration. The results suggested that nanoformulations of *O. glandulosum* essential oils are strongly recommended for therapeutic application as alternatives to antibiotics and biofilm inhibitors.

## Figures and Tables

**Table 1:** Chemical constituents of *O. glandulosum* Desf. Oil and its nanoparticles.

S/N	Compound	KI ^a^	% Area ^b^ *Origanum glandulosum* Desf. Oil			Identification Method ^c,d^
			HD	Nanoemulsion	Nanocapsules	
1	α-Thujene	928	n.d.	n.d.	1.08 ± 0.05	MS, KI, & ST
2	α-Pinene	932	0.21 ± 0.04	0.68 ± 0.02	1.73 ± 0.07	MS, KI, & ST
3	Camphene	971	n.d.	n.d.	0.39 ± 0.01	MS & KI
4	1-Octen-3-ol	973	n.d.	1.29 ± 0.13	1.64 ± 0.03	MS & KI
5	3-Octanone	981	n.d.	0.38 ± 0.05	0.58 ± 0.04	MS & KI
6	β–Myrcene	991	0.15 ± 0.03	0.79 ± 0.05	1.66 ± 0.08	MS, KI, & ST
7	α-Terpinene	1004	n.d.	0.73 ± 0.08	2.39 ± 0.1	MS, KI, & ST
8	p-Cymene	1008	27.56 ± 0.43	19.78 ± 0.16	18.58 ± 0.30	MS, KI, & ST
9	Limonene	1029	n.d.	0.64 ± 0.07	1.09 ± 0.06	MS & KI
10	Eucalyptol	1035	n.d.	n.d.	0.35 ± 0.07	MS & KI
11	γ-Terpinene	1088	5.59 ± 0.06	0.77 ± 0.09	4.52 ± 0.12	MS, KI, & ST
12	Linalool	1089	n.d.	2.98 ± 0.10	2.97 ± 0.25	MS, KI, & ST
13	Borneol	1148	n.d.	1.13 ± 0.09	2.43 ± 0.17	MS, KI, & ST
14	Terpinen-4-ol	1155	n.d.	2.73 ± 0.16	2.95 ± 0.09	MS, KI, & ST
15	α-Terpineol	1165	n.d.	1.53 ± 0.02	1.30 ± 0.12	MS & KI
16	Thymol methyl ether	1231	n.d.	0.73 ± 0.05	0.74 ± 0.05	MS & KI
17	Thymol	1267	49.52 ± 0.72	51.42 ± 0.36	39.47 ± 0.45	MS, KI, & ST
18	Carvacrol	1276	16.13 ± 0.35	9.96 ± 0.26	7.07 ± 0.10	MS, KI, & ST
19	β–Caryophyllene	1414	0.53 ± 0.02	0.78 ± 0.10	1.34 ± 0.12	MS & KI
20	α-Humulene	1451	0.12 ± 0.07	n.d.	n.d.	MS & KI
21	β–Bisabolene	1502	0.18 ± 0.07	0.89 ± 0.11	1.57 ± 0.11	MS & KI
22	β-Sesquiphellandrene	1511	n.d.	n.d.	0.65 ± 0.09	MS & KI
23	Caryophyllene oxide	1576	n.d.	1.37 ± 0.09	1.54 ± 0.14	MS & KI
	Total	-	99.99	98.58	96.04	-

^a^ Confirmed by comparison with Kovat’s index on a DB5 column [21]. ^b^ Values represent averages ± standard deviations for triplicate experiments. ^c^ Confirmed by comparison with the mass spectrum of the authentic compound. ^d^ Identification by comparison with data obtained from the NIST mass spectra library. n.d.: not detected.

**Table 2:** Average size and z-Potential of *O. glandulosum* Desf. oil nanoemulsion and nanoencapsulation.

Type of Particles	Size (nm)	Zeta Potential	PDI
Nanocapsules	120.60 ± 18.37	−15.5 ± 0.32	0.244 ± 0.05
Nanoemulsion	54.24 ± 1.29	−26.2 ± 2.6	0.296 ± 0.09

**Table 3:** Antimicrobial susceptibility patterns of MDR Gram-negative pathogens isolates against commonly used antimicrobials agents.

ATBs	Percentage of Resistance (%)									
	*Proteus* spp. (*n* = 2)	*E. cloacae* (*n* = 1)	*E. aerogene* (*n* = 1)	*P. mirabilis* (*n* = 1)	*S. marsescens* (*n* = 2)	*A. baumannii* (*n* = 2)	*M. morganii* (*n* = 2)	*E. coli* (*n* = 18)	*K. pneumoniae* (*n* = 23)	*P. aeruginosa* (*n* = 3)
AMP	100%	100%	100%	100%	100%	NT	100%	100%	100%	NT
AMX	100%	100%	100%	100%	100%	NT	100%	100%	100%	NT
AMC	100%	100%	100%	100%	100%	NT	100%	88%	91%	NT
TIC	100%	0%	100%	100%	100%	100%	50%	94%	95%	100%
PRL	100%	0%	100%	100%	100%	100%	50%	100%	95%	100%
CF	100%	75%	100%	100%	100%	NT	100%	100%	100%	NT
CTX	100%	100%	100%	100%	100%	NT	50%	88%	86%	NT
CRO	100%	25%	100%	100%	100%	NT	50%	88%	86%	NT
IMP	0%	0%	0%	100%	0%	50%	0%	5%	8%	66%
AK	100%	0%	0%	0%	0%	0%	0%	11%	0%	0%
GN	100%	0%	0%	100%	100%	50%	0%	61%	34%	100%
TCC	NT	NT	NT	NT	NT	100%	NT	NT	NT	NT
NA	0%	0%	100%	100%	50%	NT	100%	61%	56%	NT
OFX	100%	0%	0%	100%	50%	NT	100%	50%	43%	NT
CIP	100%	0%	0%	100%	50%	NT	50%	55%	39%	NT
TZP	NT	NT	NT	NT	NT	100%	NT	NT	NT	33%
CT	100%	0%	0%	100%	100%	0%	100%	5%	0%	33%
SXT	100%	75%	100%	50%	100%	50%	100%	77%	91%	100%
ATM	NT	NT	NT	NT	NT	50%	NT	NT	NT	33%
CAZ	NT	NT	NT	NT	NT	100%	NT	NT	NT	100%
LEV	NT	NT	NT	NT	NT	0%	NT	NT	NT	66%
RD	NT	NT	NT	NT	NT	0%	NT	NT	NT	33%
NET	NT	NT	NT	NT	NT	0%	NT	NT	NT	66.66%

ATBs: Antibiotics, NT: not tested, AMP: Ampicillin, AMX: Amoxicillin, AMC: Amoxicillin/clavulanate TIC: Ticarcillin, PRL: Piperacillin, CF: Cefazolin, CTX: Cefotaxime, CRO: Ceftriaxone, IMP: Imipenem, GN: Gentamicin, OFX: Ofloxacin, CIP: Ciprofloxacin, CT: Colistin, SXT: Trimethoprim/Sulfamethoxazole, AK: Amikacin, TCC: Ticarcillin-clavulanate, TZP: Piperacillin-clavulanate, CAZ: Ceftazidime, ATM: Aztreonam, LEV: Levofloxacin, NET: Netlmicin, RD: Rifampicin, NA: Nalidixic acid.

**Table 4:** Antimicrobial susceptibility patterns of *Methicillin-resistant S. aureus* against antimicrobial agents.

Antibiotics (ATBs)	Resistance Percentage (%) MRSA (*N* = 14)
P	100%
OX	100%
FOX	100%
E	21%
SP	14%
L	14%
DA	14%
PT	7%
VA	0%
AK	28%
GN	42%
OFX	21%
FD	42%
TEC	0%
TE	42%
DO	14%
C	0%
RD	7%

P: Penicillin, OX: Oxacillin, FOX: Cefoxitin, SP: Spriramycin, DA: Clindamycin, PT: Pristinamycin, L: Lincomycin, E: Erythromycin, RD: Rifampicin, VA: Vancomycin, AK: Amikacin, OFX: Ofloxacin, TEC: Teicoplanin, TE: Tetracycline, DO: Doxycycline, FD: Fusidicacid, C: Champenicol, GN: Gentamicin.

**Table 5:** Antibacterial activity of *O. glandulosum* HD oil and its nanoparticles against multidrug resistant isolates using three methods (Aromatogram, MIC, and MBC).

Strains	*O. glandulosum oil*			*Nanoemulsion*			*Nanoencapsulation*		
	IZ * (mm)	MIC (%)	MBC (%)	IZ (mm)	MIC (µL/mL)	MBC (µL/mL)	IZ (mm)	MIC (µL/mL)	MBC (µL/mL)
*E. coli ESBL*	13	0.62%	0.62%	NA **	/	/	11	31.25	31.25
*K. pneumoniae* *ESBL*	15	0.62%	1.25%	NA	/	/	NA	/	/
*A. baumannii*	40	0.07%	0.15%	16	62.5	125	11	31.25	31.25
*S. marcescens* *ESBL*	16	0.31%	0.31%	NA	/	/	11	31.25	125
*MRSA*	35	0.15%	0.15%	12	62.5	62.5	13	31.25	62.5
*P. aeruginosa* *ATCC27853*	NA	/	/	NA	/	/	NA	/	/
*E. coli ATCC* *25953*	20	0.15%	0.15%	12	62.5	125	20	31.25	250
*K. pneumoniae ATCC700603*	17	0.15%	0.15%	15	31.25	125	20	31.25	250
*S. aureus ATCC* *6538P*	22	0.15%	0.15%	20	31.25	62.5	20	31.25	125

* IZ: inhibition zone, ** NA: not active.

## Data Availability

The data presented in this study are available in this article.
